# Supplementary material for: The Treatment Effect of Liver Transplantation versus Liver Resection for HCC: A Review and Future Perspectives
Source: Cancers (Basel). 2021 Jul 24;13(15):3730. doi: 10.3390/cancers13153730 (PMC8345205; doi:10.3390/cancers13153730)
Supplement: Supplementary file 1 [file cancers-13-03730-s001.zip › Supplementary data 1 Search terms.pdf]

cancers

MDPI

The Treatment Effect of Liver Transplantation versus Liver Resection for HCC: A Review and Future Perspec-tives

Berend R. Beumer, Roeland F. de Wilde, Herold J. Metselaar, Robert A. de Man, Wojciech G. Polak and Jan N.M. IJzermans

Search systematic reviews: LT vs. LR for early HCC

Database	Number of references
Embase	652
Web of science	245
Cochrane central	287
Total	1184
Total deduplicated	1010

Embase: 652

('liver tumor'/de OR 'liver cancer'/de OR 'liver carcinogenesis'/exp OR 'liver cell carcinoma'/exp OR 'hepatobiliary system tumor'/de OR ((liver OR hepat* OR intrahepat*) NEAR/3 (cancer OR carcino* OR neoplas* OR tumo* OR malign* OR lesion*) OR hcc):ab,ti) AND ('liver transplantation'/exp OR (((liver OR hepat*) AND (transplant* OR graft*))):kw,de,ab,ti) AND ('liver resection'/exp OR hepatectom* OR ((hepat* OR liver) AND (resect* OR 'surgery'/exp OR 'surgery':lnk OR (surg* OR operative* OR operation* OR resect*):ab,ti))) AND ((('systematic' AND 'review') OR ('meta' AND 'analysis')):ab,ti OR 'systematic review'/exp)

Web of science: 245

(TS=((((liver OR hepat* OR intrahepat*) NEAR/2 (cancer OR carcino* OR neoplas* OR tumo* OR malign* OR lesion*) OR hcc)) AND ((((liver OR hepat* OR organ OR organs) AND (transplant* OR graft* OR allotransplant* OR allograft* OR autotransplant* OR autograft* OR recipient*)))) AND ((surg* OR operative* OR operation* OR resect* OR hepatectom*)))) AND (ALL=((review AND systematic) OR (meta AND analysis)))

Cochrane CENTRAL: 287 # no restriction on article type

(((liver OR hepat* OR intrahepat*) NEAR/3 (cancer OR carcino* OR neoplas* OR tumo* OR malign* OR lesion*) OR hcc):ab,ti) AND ((((liver OR hepat* OR organ OR organs) AND (transplant* OR graft* OR allotransplant* OR allograft* OR autotransplant* OR autograft* OR recipient*))):ab,ti) AND ((surg* OR operative* OR operation* OR resect* OR hepatectom*):ab,ti)

Google scholar

"liver|hepatic|intrahepatic cancer|carcinoma|neoplasm|tumor|malignancy|lesion" AND "liver|hepatic transplantation|graft|recipient" AND "resection|hepatectomy|hepatectomies" AND "review|meta"

Articles after last systematic review (≥ 2017) LT vs. LR for early HCC

Database	Number of references
Embase	963
Web of science	814
Cochrane central	235
Total	1248
Total deduplicated	1159

Embase 963

('liver tumor'/mj OR 'liver cancer'/mj OR 'liver carcinogenesis'/mj/exp OR 'liver cell carcinoma'/mj/exp OR ((liver OR hepat* OR intrahepat*) NEAR/3 (cancer OR carcino* OR neoplas* OR tumo* OR malign* OR lesion*) OR hcc):ti) AND ('liver transplantation'/mj/exp OR (((liver OR hepat*) AND (transplant* OR graft*))):ti) AND ('liver resection'/mj/exp OR hepatectom* OR ((hepat* OR liver) AND (resect* OR 'surgery'/mj/exp OR 'surgery':lnk OR (surg* OR operative* OR operation* OR resect*):ti))) AND (2017:py OR 2018:py OR 2019:py OR 2020:py OR 2021:py) NOT ((('systematic' AND 'review') OR ('meta' AND 'analysis')):ab,ti OR 'systematic review'/exp OR 'case series'/exp OR 'case series' OR (case AND series)) AND ('randomized controlled trial'/exp OR 'cohort analysis'/exp OR 'longitudinal study'/de OR 'prospective study'/de OR 'retrospective study'/de OR ((random* NEAR/3 trial*) OR cohort* OR longitudinal* OR prospective* OR retrospective*):ab,ti)

Web of science: 814

((AB=((((liver OR hepat* OR intrahepat*) NEAR/2 (cancer OR carcino* OR neoplas* OR tumo* OR malign* OR lesion*) OR hcc)) AND ((((liver OR hepat* OR organ OR organs) AND (transplant* OR graft* OR allotransplant* OR allograft* OR autotransplant* OR autograft* OR recipient*)))) AND ((surg* OR operative* OR operation* OR resect* OR hepatectom*))))) AND (PY==("2017" OR "2018" OR "2019" OR "2020" OR "2021") AND DT==("ARTICLE") AND LA==("ENGLISH"))

Cochrane CENTRAL: 235

(((liver OR hepat* OR intrahepat*) NEAR/3 (cancer OR carcino* OR neoplas* OR tumo* OR malign* OR lesion*) OR hcc):ab,ti) AND ((((liver OR hepat* OR organ OR organs) AND (transplant* OR graft* OR allotransplant* OR allograft* OR autotransplant* OR autograft* OR recipient*))):ab,ti) AND ((surg* OR operative* OR operation* OR resect* OR hepatectom*):ab,ti)

With publication date limited to: 1-01-2017 to 01-07-2021
