# Supplementary material for: The Treatment Effect of Liver Transplantation versus Liver Resection for HCC: A Review and Future Perspectives
Source: Cancers (Basel). 2021 Jul 24;13(15):3730. doi: 10.3390/cancers13153730 (PMC8345205; doi:10.3390/cancers13153730)
Supplement: Supplementary file 1 [file cancers-13-03730-s001.zip › Supplementary data 2 Table meta-analysis DFS.pdf]

cancers

The Treatment Effect of Liver Transplantation ver-sus Liver Resection for HCC: A Review and Future Perspectives

Berend R. Beumer, Roeland F. de Wilde, Herold J. Metselaar, Robert A. de Man, Wojciech G. Polak and Jan N. M. Ijzermans

Table S1. – Meta-analyses of systematic reviews (DFS).

Author	Year of Publication	Population	5-year DFS					
			Number of studies	Number of patients	Pooled OR (95%CI)	*p*-value	I^2	Conclusion
Non Intention-To-Treat								
Rahman [1]	2012	Non-ITT	7	1494	0.29 (0.20-0.42)	<0.001	56	In favour of LT
Dhir [2]	2012	Within MC	-	-	-	-	-	-
Dhir [2]	2012	Within MC. Well compensated cirrho-sis	-	-	-	-	-	-
Schoenberg [3]	2017	Within MC. No Child-Pugh class C	-	-	-	-	-	-
Xu [4]	2014		12	3564	0.18 (0.14–0.23) *	-	40	In favour of LT
Zheng [5]	2014		23	5753	0.18 (0.13–0.24) *	<0.001	76	In favour of LT
Intention-to-treat								
Rahman [1]	2012	ITT	3	997	0.76 (0.57-1.00)	0.05	0	In favour of LT
Dhir [2]	2012	ITT. Within MC	-	-	-	-	-	-
Dhir [2]	2012	ITT. Within MC. Well compensated cirrhosis	-	-	-	-	-	-
Proneth [6]	2014	ITT	-	-	-	-	-	-
Menahem [7]	2017	ITT	7	1080	0.18 (0.06–0.53)		91	In favour of LT

Overview of the meta-analyses from systematic reviews for disease free survival (DFS). All systematic reviews included studies that described patients with hepatocellular carcinoma receiving liver resection or liver transplantation as first line therapy. Additional criteria defining the study population are listed in the table. Odds were calculated as the number of deaths or recurrences divided by number of patients alive and without recurrence. Odds ratio (OR) was calculated as liver transplantation odds divided by liver resection odds. Note: * To set the reference group to liver resection the odds ratio reported in the systematic review was inverted. Abbreviations: patients (pt). Child-Pugh class (CP). Intention-to-treat (ITT). Milan Criteria (MC).

References

1. Rahman, A.; Assifi, M.M.; Pedroso, F.E.; Maley, W.R.; Sola, J.E.; Lavu, H.; Winter, J.M.; Yeo, C.J.; Koniaris, L.G. Is resection equivalent to transplantation for early cirrhotic patients with hepatocellular carcinoma? A meta-analysis. *J. Gastrointest. Surg.* **2012**, *16*, 1897–1909.
2. Dhir, M.; Lyden, E.R.; Smith, L.M.; Are, C. Comparison of outcomes of transplantation and resection in patients with early hepatocellular carcinoma: A meta-analysis. *HPB* **2012**, *14*, 635–645.
3. Schoenberg, M.B.; Bucher, J.N.; Vater, A.; Bazhin, A.V.; Hao, J.; Guba, M.O.; Angele, M.K.; Werner, J.; Rentsch, M. Resection or transplant in early hepatocellular carcinoma: A systematic review and meta-analysis. *Dtsch. Ärzteblatt Int.* **2017**, *114*, 519.
4. Xu, X.-S.; Liu, C.; Qu, K.; Song, Y.-Z.; Zhang, P.; Zhang, Y.-L. Liver transplantation versus liver resection for hepatocellular carcinoma: A meta-analysis. *Hepatobiliary Pancreat. Dis. Int.* **2014**, *13*, 234–241.
5. Zheng, Z.; Liang, W.; Milgrom, D.P.; Zheng, Z.; Schroder, P.M.; Kong, N.S.; Yang, C.; Guo, Z.; He, X. Liver transplantation versus liver resection in the treatment of hepatocellular carcinoma: A meta-analysis of observational studies. *Transplantation* **2014**, *97*, 227–234.
6. Proneth, A.; Zeman, F.; Schlitt, H.J.; Schnitzbauer, A.A. Is resection or transplantation the ideal treatment in patients with hepatocellular carcinoma in cirrhosis if both are possible? A systematic review and metaanalysis. *Ann. Surg. Oncol.* **2014**, *21*, 3096–3107.

7. Menahem, B.; Lubrano, J.; Duvoux, C.; Mulliri, A.; Alves, A.; Costentin, C.; Mallat, A.; Launoy, G.; Laurent, A. Liver transplantation versus liver resection for hepatocellular carcinoma in intention to treat: An attempt to perform an ideal meta-analysis. *Liver Transplant.* **2017**, *23*, 836–844.
